# Methanobacterium nebraskense sp. nov., a hydrogenotrophic methanogen isolated from saline wetland soil

**DOI:** 10.1099/ijsem.0.007139

**Published:** 2026-04-21

**Authors:** Nicole A. Fiore, You Zhou, Karrie A. Weber

**Affiliations:** 1 School of Biological Sciences, University of Nebraska–Lincoln, Lincoln, Nebraska, USA; 2 Center for Biotechnology, University of Nebraska–Lincoln, Lincoln, Nebraska, USA; 3 Department of Earth and Atmospheric Sciences, University of Nebraska–Lincoln, Lincoln, Nebraska, USA; 4 Daugherty Water for Food Institute, University of Nebraska, Lincoln, Nebraska, USA

**Keywords:** archaea, methane, methanogen, methanogenesis, *Methanobacterium*, wetland

## Abstract

A hydrogenotrophic methanogen, designated strain ACI-7^T^, was isolated from an alkaline, saline wetland located in eastern Nebraska, USA. Cells were identified as non-motile rods (0.9–4.2 µm in length and 0.2–0.4 µm in diameter) that occurred singly, in chains, or as twisted chains and aggregates. Strain ACI-7^T^ utilized H_2_+CO_2_ or formate as methanogenic substrates and required growth factors present in yeast extract for continuous cultivation. The strain grew at 20–45 °C (optimum, 40 °C), at pH 6.5–8.5 (optimum, pH 7.3) and with 0–2.5% NaCl (optimum, 0–1%). The genomic G+C content of ACI-7^T^ was 31.79 mol%. Phylogenetic analysis of the 16S rRNA gene sequence indicated strain ACI-7^T^ was affiliated with the genus *Methanobacterium*, most closely related to *Methanobacterium oryzae* FPi^T^ (97.1% sequence similarity) and *Methanobacterium veterum* MK4^T^ (96.9% sequence similarity). Overall genome relatedness indices for strain ACI-7^T^ compared to other *Methanobacterium* species ranged from 68.45 to 78.17% for average nucleotide identity and 18.9 to 25.7% for digital DNA–DNA hybridization. Morphological, physiological and genomic characteristics indicate that strain ACI-7^T^ represents a novel species, for which the name *Methanobacterium nebraskense* sp. nov. is proposed. The type strain is ACI-7^T^ (=DSM 118696^T^=ATCC TSD-487^T^).

## Introduction

Hydrogenotrophic methanogenesis is an evolutionarily ancient [1,2] microbial metabolism by which carbon dioxide is reduced to methane using hydrogen as an electron donor. Of the known methanogenic pathways, hydrogenotrophic methanogenesis is also the most widespread, distributed across several taxonomic phyla within the domain Archaea [1,3]. Some of the earliest hydrogenotrophic methanogens studied were associated with the genus *Methanobacterium* [4,8], which currently includes 27 species with validly published names [9,33] according to the List of Prokaryotic names with Standing in Nomenclature (LPSN) [34]. *Methanobacterium* species have been isolated from several sites, including terrestrial [13,33], marine [9] and subsurface [22,25, 30] environments. Five species have been isolated from wetland ecosystems: *Methanobacterium paludis* [27] and *Methanobacterium palustre* [28] from peatlands, *Methanobacterium uliginosium* [32] from a marsh and *Methanobacterium kanagiense* [23] and *Methanobacterium oryzae* [26] from rice fields. In this study, we describe a novel *Methanobacterium* species, strain ACI-7^T^, isolated from a wetland located within Nebraska’s eastern saline wetland complex. The wetlands within this system are described as saline and alkaline, though geochemical parameters vary both spatially and seasonally in response to hydrologic conditions (e.g. groundwater discharge, precipitation, evaporation, etc.) [35,36]. Strain ACI-7^T^ represents the first published microbial isolate from this regionally unique wetland environment.

### Enrichment and isolation

Wetland soil samples were collected in June 2013 from an alkaline, saline wetland in Lincoln, NE, USA (40° 52′ 43.0″ N 96° 40′ 35.8″ W) as described previously [37]. Initial enrichments were performed with 50% w/v wetland soil in Nebraska Saline Wetland (NSW) culture medium [37] amended with calcium carbonate as a sole source of inorganic carbon and hydrogen as an electron donor. The composition of the initial enrichment medium (NSW) was 0.6 g NaH_2_PO_4_, 0.25 g NH_4_Cl, 0.38 g KCl, 4.77 g HEPES, and 1% v/v each of vitamin and trace mineral solution [38] per litre of double deionized water. The medium was adjusted to pH 7.7 with 10 M NaOH, heated and sparged with oxygen-free nitrogen gas (100% N_2_) and autoclaved at 121 °C for 20 min before amendment with calcium carbonate (0.01 g ml^−1^) and hydrogen gas (0.1 ml H_2_ per millilitre liquid volume). After several successive transfers (10% v/v), genome-resolved metagenomic analysis of the initial enrichment community identified five bacterial taxa and one methanogen [37]. Metabolic pathways and antibiotic resistance genes identified in the metagenomic dataset were used to develop culture conditions that would select for the methanogen while suppressing the growth of the bacterial community members. The methanogen was subsequently enriched from the initial community using bicarbonate-buffered (100 mM) MS Enrichment Medium [39], prepared and dispensed under Ar/CO_2_ (80:20 v/v) using Hungate technique [40,42] and autoclaved at 121 °C for 20 min. After autoclaving, sodium sulphide (1 mM), sodium mercaptoethanesulphonate (coenzyme M; 3 mM) and antibiotics (100 µg ml^−1^ kanamycin, 100 µg ml^−1^ clindamycin, 100 µg ml^−1^ tetracycline and 250 µg ml^−1^ vancomycin) were added to the medium from sterile anoxic stock solutions, with a final medium pH of 7.3. After inoculation (10% v/v), hydrogen gas was added via syringe (20 ml H_2_ overpressure for 10 ml culture in 26 ml Balch tubes). Cultures were incubated statically at 37 °C in the dark, positioned horizontally to maximize surface area for gas exchange.

Pure cultures were obtained by isolating single colonies from agar shake tubes [43] in an anaerobic chamber. One of these cultures was given the strain designation ACI-7 and characterized further. Antibiotics were omitted for subsequent transfers in liquid medium (5% v/v), including those used to evaluate culture purity. Culture purity was confirmed based on microscopic observation, absence of growth with methanogenic substrates omitted and failed PCR amplification using high coverage, bacteria-specific 16S rRNA primer pairs S-D-Bact-0008-a-S-16/S-D-Bact-1492-a-A-16 and S-D-Bact-0341-b-S-17/S-D-Bact-1061-a-A-17 [44] relative to positive controls. Pure cultures were stored at −80 °C in anoxic 10% (v/v) glycerol for long-term preservation.

### Morphology and cellular observations

Cells for microscopy were grown to mid-log phase on MS Enrichment Medium with H_2_ and CO_2_ as substrates. Cell morphology, size, motility and arrangement were observed using a combination of phase-contrast microscopy, differential interference contrast (DIC) microscopy, fluorescence microscopy and scanning electron microscopy. Phase-contrast microscopy was performed on unfixed samples using an Axioskop 40 FL microscope (Zeiss) equipped with an AxioCam ERc 5s (Zeiss). DIC microscopy and fluorescence microscopy were conducted using a Nikon A1R-Ti2 confocal laser scanning microscope on cells fixed with 2.5% glutaraldehyde in 0.1 M cacodylate buffer, rinsed twice with the same buffer and once with 10% ethanol and then stained with 10 µM SYTOX Green. Cell length and diameter were estimated from phase-contrast and fluorescence micrographs using ImageJ2. F_420_ autofluorescence [45] was examined with an Axioskop 40 FL microscope equipped with an HBO 50 fluorescence illuminator (Zeiss) and an F420 filter set (417/60 nm excitation, 482/35 nm emission, 458 nm beam splitter; AVR Optics). For scanning electron microscopy, cells were fixed with 2.5% glutaraldehyde in 0.1 M cacodylate buffer, ethanol dehydrated, air dried overnight and then sputter coated with a thin layer (~2 nm) of chromium. The coated samples were examined under a field-emission scanning electron microscope (S-4700, Hitachi). Susceptibility to lysis by SDS and distilled water were determined as described by Boone and Whitman [46], with cell lysis assessed via OD and phase-contrast microscopy.

**Fig. 1.**
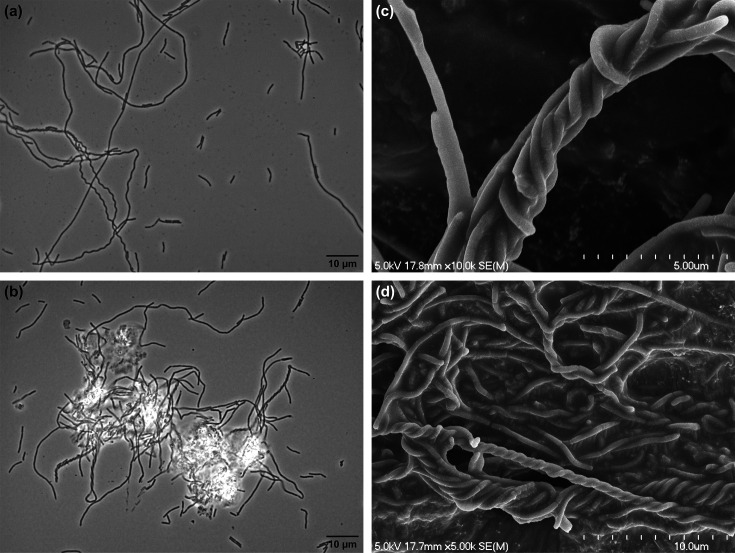
Cell morphology of *Methanobacterium nebraskense* strain ACI-7^T^ as observed with phase-contrast microscopy (**a, b**) and scanning electron microscopy (**c, d**). Cells occur singly, in chains or as twisted chains and aggregates. Cultures were grown with H_2_+CO_2_ under optimal conditions. Scale bars represent 10 µm (**a, b, d**) and 5 µm (**c**).

Cells of strain ACI-7^T^ are short rods, 0.9–4.2 µm in length and 0.2–0.4 µm in diameter, occurring as single cells, chains, long filaments and aggregates (Figs1  2). Single cells and straight chains are common in early cultures, but as cell density increases, aggregates are observed more frequently. Some chains form twisted, helical structures, either as single chains that twist upon themselves (forming a loop at one end) (Fig. 1b) or multiple chains twisted together (Fig. 1c, d), similar to morphological observations described for *Methanobacterium aggregans* [10]. Cells are non-motile and stain Gram-negative. Strong autofluorescence is observed at 420 nm (Fig. 3) but rapidly fades. Cells resist lysis by both 0.1 and 1% SDS and by hypotonic solution (distilled water).

**Fig. 2.**
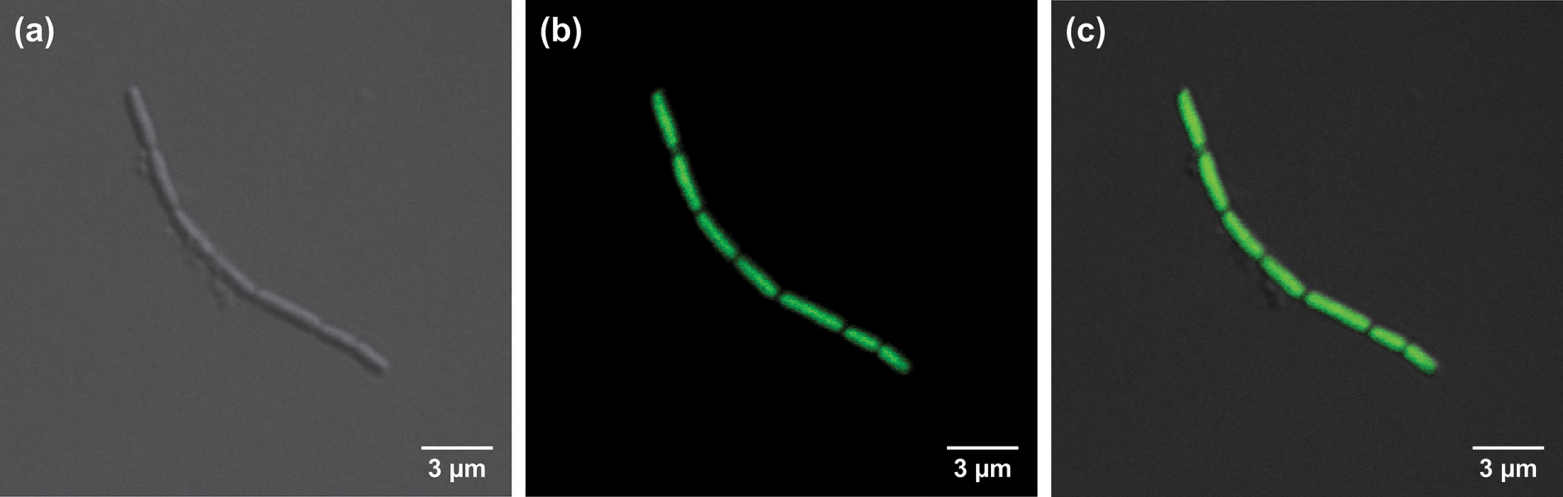
Photomicrographs of *Methanobacterium nebraskense* strain ACI-7^T^ obtained with DIC microscopy (**a**) and fluorescence microscopy of SYTOX-stained cells (**b**), along with an overlay of both images (**c**). Cells were grown on H_2_+CO_2_ under optimal conditions. Scale bars represent 3 µm.

**Fig. 3.**
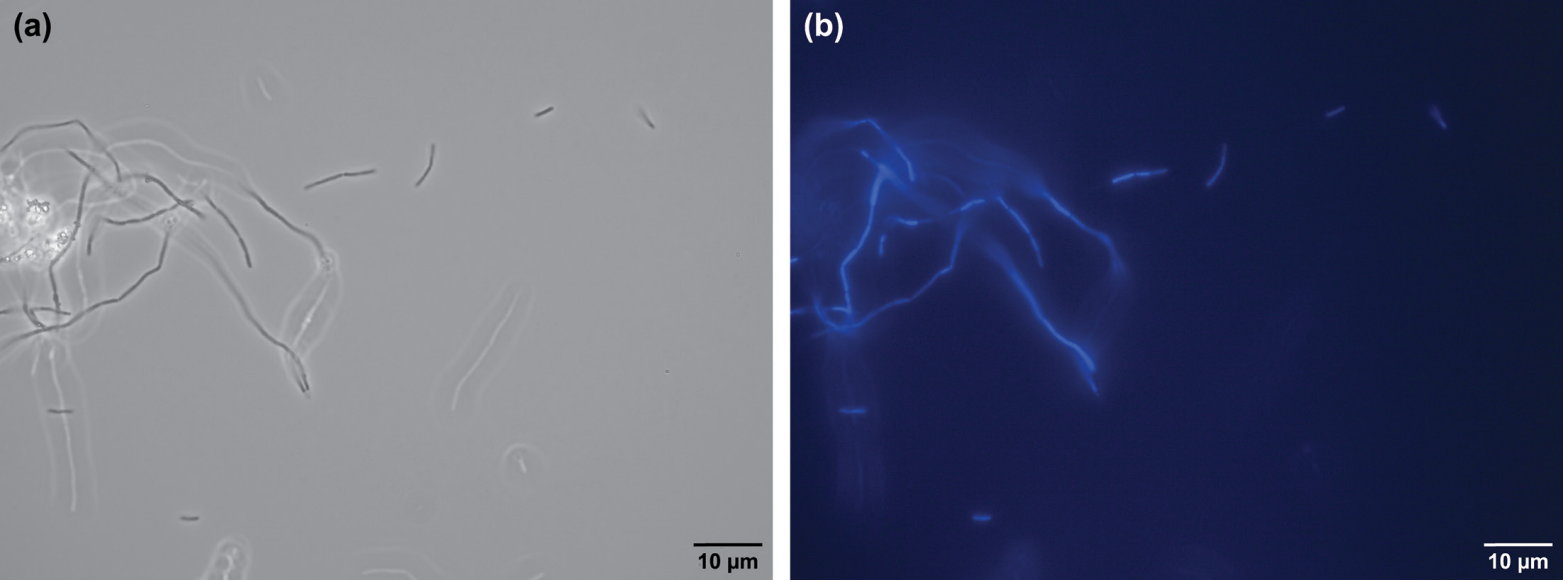
Phase-contrast (**a**) and fluorescence (**b**) photomicrographs showing F_420_ autofluorescence by cells of *Methanobacterium nebraskense* strain ACI-7^T^. Cells were grown on H_2_+CO_2_ under optimal conditions. Scale bars represent 10 µm.

### Substrate utilization and growth conditions

Growth and substrate utilization were assessed by culturing strain ACI-7^T^ in MS Enrichment Medium on H_2_+CO_2_ at 37 °C as described above, unless otherwise noted. All tests were performed in triplicate, and growth was monitored by measuring the concentration of methane in the headspace [47]. Methane was measured by GC with a Varian 430-GC and thermal conductivity detector using ultra high-purity argon as carrier gas, with oven and detector temperatures of 65 and 250 °C, respectively.

**Fig. 4.**
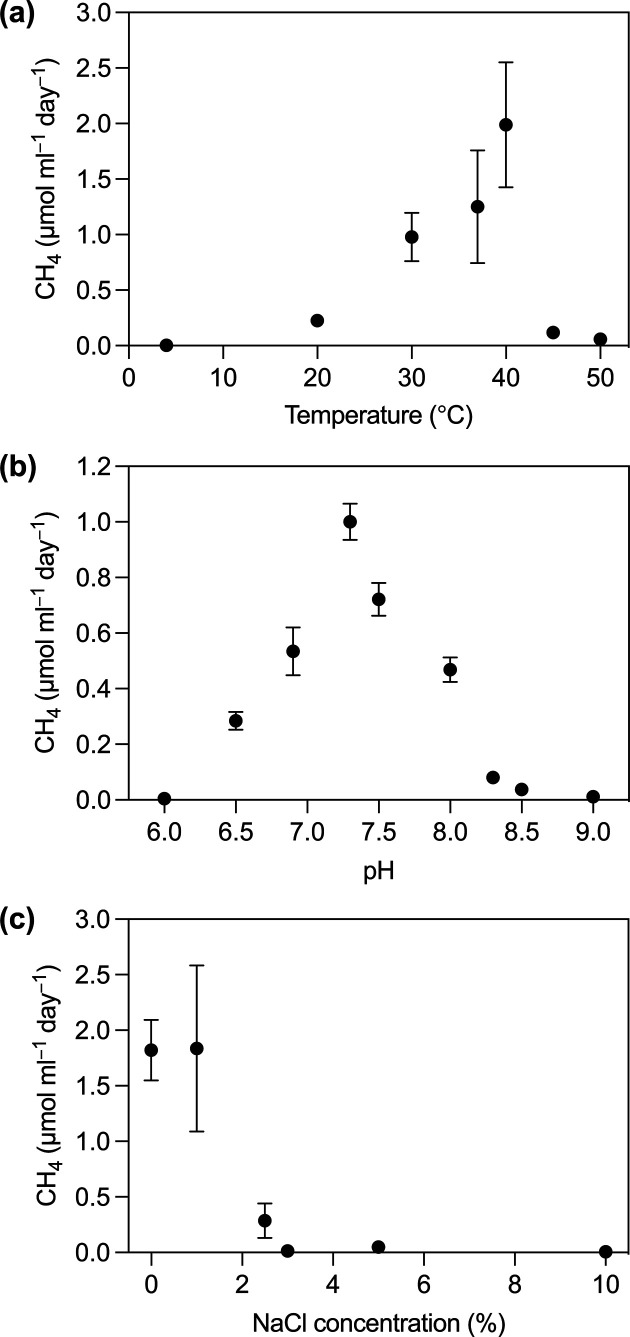
Influence of temperature (**a**), pH (**b**) and NaCl concentration (**c**) on growth of strain ACI-7^T^. Growth is represented by the maximum observed rate of methane production with H_2_+CO_2_ as substrates. Values are reported in µmol CH_4_ produced per millilitre culture per day. Error bars represent the standard error of measurement for triplicate cultures; error bars not visible are smaller than the symbol.

The effect of temperature on growth was tested at 4, 20, 30, 37, 40, 45 and 50 °C. Incubations from 30 to 50 °C were accomplished with separate incubators at their respective temperatures, 20 °C on the bench at room temperature and 4 °C in a laboratory refrigerator. Growth of strain ACI-7^T^ was observed from 20 to 45 °C (optimum 40 °C), with no growth at 4 or 50 °C (Fig. 4a). The effect of NaCl on growth was tested with 0, 1, 2.5, 3, 3.5, 4.5, 5 and 10% NaCl (w/v). Medium was prepared with 0 and 10% NaCl and mixed prior to dispensing to achieve intermediate concentrations. Growth was observed with NaCl concentrations from 0 to 2.5%, with optimum growth from 0 to 1%, and no growth observed at 3% NaCl (Fig. 4b). The effect of pH on growth was measured at pH values of 6.0, 6.5, 6.9, 7.3, 7.5, 8.0, 8.3, 8.5 and 9.0. The pH of MS Enrichment Medium as described above is 7.3; to achieve other values, the medium was supplemented with organic buffers, as recommended for bicarbonate-buffered media [48]. The organic buffers used were as follows, all at 50 mM concentrations: pH 6.0 (MES), pH 6.5–6.9 (PIPES), pH 7.5–8.5 (HEPES), and pH 9.0 (CHES). Adjustments to pH were made with sterile, anoxic stock solutions of 1 M HCl or 1 M Na_2_CO_3_ prior to inoculation. pH was measured immediately after inoculation and increased by no more than 0.2 units after incubation. Growth of strain ACI-7^T^ was observed over pH 6.5–8.5 (optimum 7.3), with no growth at pH 6.0 or 9.0 (Fig. 4c).

To determine nutrient requirements, all sources of organic carbon (yeast, tryptone and coenzyme M) were omitted from the medium. Growth of strain ACI-7^T^ was tested in minimal medium alone and with the following additions: yeast extract (0.5 g l^−1^), tryptone (0.5 g l^−1^), casamino acids (0.5 g l^−1^), acetate (10 mM), coenzyme M (3 mM) and Wolfe’s vitamin solution [49]. Growth was monitored for six successive transfers at 5% (v/v) inoculum. An unidentified component of yeast extract was required for stable cultivation of strain ACI-7^T^; casamino acids, acetate and vitamins were not adequate substitutes. Coenzyme M alone was sufficient for four successive transfers, but methane production declined with the fifth and sixth transfer.

The following compounds were tested as potential substrates for methanogenesis: H_2_+CO_2_, formate, acetate, dimethylsulphide, methanol, 2-propanol+CO_2_, H_2_+methanol, H_2_+methylamine, methylamine, dimethylamine and trimethylamine. All aqueous substrates were tested at concentrations of 10 mM, with positive results confirmed by three successive transfers and compared to no-substrate controls. When testing growth on substrates other than carbon dioxide in combination with hydrogen (e.g. H_2_+methanol, H_2_+methylamine), the bicarbonate buffer was replaced with 50 mM HEPES, and medium was prepared and dispensed under 100% Ar headspace. H_2_+CO_2_ and formate were the only substrates used for growth and methanogenesis, with specific growth rates of 0.033 and 0.016 h^−1^ under optimal conditions, respectively. Acetate, dimethylsulphide, methanol, 2-propanol+CO_2_, H_2_+methanol, H_2_+methylamine, methylamine, dimethylamine or trimethylamine were not used as substrates for methane production.

### Genome and phylogenetic classification

The genome sequence for strain ACI-7^T^ was obtained as previously described [50]. Briefly, DNA was extracted using a method modified from Zhou *et al*. [51], and library preparation and sequencing (PacBio Sequel IIe) were completed by the University of Delaware DNA Sequencing and Genotyping Center. Reads were assembled with Flye 2.9.1 [52], resulting in a single, circular contig 2,239,949 bp in length at an estimated 5,253× coverage with a G+C content of 31.79 mol% [50]. The genome was 99.2% complete with no contamination, based on conserved single-copy genes for Euryarchaeota using CheckM v1.1.2 [53]. Genome annotation with the NCBI Prokaryotic Genome Annotation Pipeline (PGAP) 6.8 [54] predicted 2,411 genes representing 2,360 protein-coding sequences, 2 complete sets of rRNAs (5S, 16S and 23S rRNAs), 37 tRNAs and 2 noncoding RNAs (ncRNAs). The two 16S rRNA gene sequences were extracted from the genome assembly using Barrnap 0.9 (https://github.com/tseemann/barrnap), both of which were 1,478 bp in length with 100% identity. Additional annotation was performed with BlastKOALA against the KEGG GENES database [55]. Genomic evidence for hydrogenotrophic methanogenesis in ACI-7^T^ includes all necessary genes for the Wolfe cycle of carbon dioxide reduction to methane [56] and the archaeal type Wood-Ljungdahl pathway of carbon fixation [57] (Table S2, available in the online Supplementary Material).

To confirm the authenticity of the genome assembly, a partial 16S rRNA gene sequence was obtained via Sanger sequencing for comparison to the genome-derived sequences [58]. The 16S rRNA gene was PCR amplified from strain ACI-7^T^ using the primers A109F [59] and Met1340R [60] and sequenced by Eurofins Genomics (Louisville, KY), resulting in a partial 16S rRNA gene sequence 1,045 bp in length. The 1,045 bp 16S rRNA gene sequence obtained via Sanger sequencing was identical to the full-length gene sequence obtained from the genome, with no gaps or mismatches. The full-length 1,478 bp 16S rRNA gene sequence extracted from the genome assembly was used for phylogenetic analysis.

Both phylogenetic (16S rRNA) and phylogenomic analyses were used to infer the taxonomy and phylogeny of strain ACI-7^T^. Phylogenetic analysis based on the 16S rRNA gene was performed for strain ACI-7^T^ with the type strain of all *Methanobacterium* species with validly published names (Table S1). The 16S rRNA sequences were obtained from GenBank and aligned with muscle 5.1 [61]. The alignment was trimmed with BMGE [62], and maximum-likelihood phylogenetic trees were inferred using RAxML v8.2.12 [63] using 1,000 bootstrap replicates. Pairwise sequence similarities were estimated using the EzBioCloud server [64]. At the genomic level, overall genome relatedness indices (OGRIs) and phylogenomic trees were determined for strain ACI-7^T^ against all *Methanobacterium* spp. with available reference genomes (Table S1). Genomes were obtained from NCBI RefSeq or JGI IMG, with preference for type strains when available (Table S1). For overall genome relatedness, average nucleotide identity (ANI) and digital DNA–DNA hybridization (dDDH) were calculated using OrthoANIu [65] and the Genome-to-Genome Distance Calculator (GGDC) 3.0 with Formula 2 [66], respectively. Maximum-likelihood phylogenomic trees were reconstructed based on 53 conserved archaeal marker genes obtained from the Genome Taxonomy Database (GTDB) R226 [67] using PhyloPhlAn 3.1 [68] and RAxML-NG v1.2.0 [69] with 100 bootstrap replicates.

**Fig. 5.**
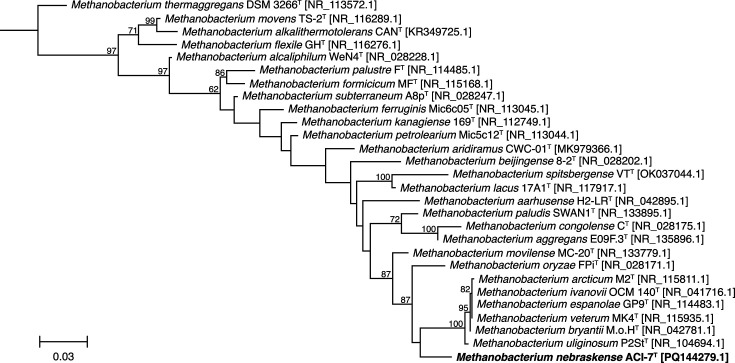
Maximum-likelihood phylogenetic tree based on 16S rRNA gene sequences (1,326 bp) showing the position of strain ACI-7^T^ and type strains of the genus *Methanobacterium*. Numbers at nodes indicate bootstrap percentages based on 1,000 replicates; only values ≥50% are shown. Accession numbers are indicated in brackets. *Methanothermus fervidus* V24S^T^ [NR102926.1] was used as an outgroup (not shown). Bar, 0.03 substitutions per nucleotide position.

**Fig. 6.**
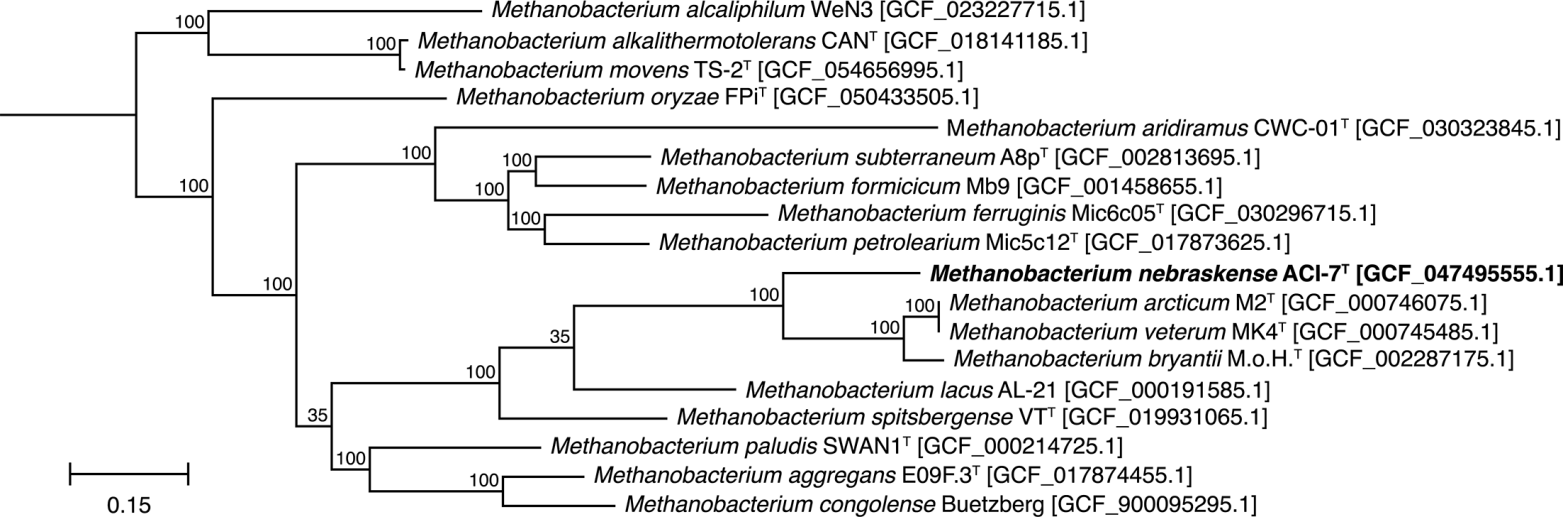
Maximum-likelihood phylogenomic tree of strain ACI-7^T^ in relation to other *Methanobacterium* species with available reference genomes. Trees were inferred based on the 53 conserved archaeal marker genes in the GTDB. Values at nodes indicate bootstrap percentages based on 100 replicates. Accession numbers are indicated in brackets. *Methanothermus fervidus* V24S^T^ [GCF_000166095.1] was used as an outgroup (not shown). Bar, 0.15 substitutions per nucleotide position.

Phylogenetic trees reconstructed from 16S rRNA gene sequences indicated strain ACI-7^T^ falls within the genus *Methanobacterium*, forming a cluster including *M. oryzae* FPi^T^ and a clade containing *Methanobacterium uliginosum* P2S^T^, *Methanobacterium bryantii* M.o.H.^T^, *Methanobacterium arcticum* M2^T^, *Methanobacterium espanolae* GP9^T^, *Methanobacterium ivanovii* OCM 140^T^ and *Methanobacterium veterum* MK4^T^ (Fig. 5). Based on 16S rRNA gene similarity, strain ACI-7^T^ was most closely related to * M. oryzae* FPi^T^ (97.09% sequence similarity), followed by *M. veterum* MK4^T^ (96.95% sequence similarity), *M. ivanovii* OCM 140^T^ (96.86% sequence similarity) and *Methanobacterium movilense* MC-20^T^ (96.73% sequence similarity), all of which fall below the recommended species-level cutoff of 98.65% [70]. At the phylogenomic level, strain ACI-7^T^ formed a highly supported cluster with the clade containing *M. bryantii* M.o.H.^T^, *M. arcticum* M2^T^ and *M. veterum* MK4^T^ (Fig. 6), but not with *M. oryzae* FPi^T^ as shown in the 16S rRNA gene tree. *M. oryzae* FPi^T^, the closest relative to strain ACI-7^T^ based on 16S rRNA sequence similarity, also had the highest ANI. Compared with strain ACI-7^T^, *M. oryzae* FPi^T^ had an ANI value of 78.17%, followed by *M. arcticum* M2^T^ and *M. bryantii* M.o.H.^T^ with values of 75.56 and 75.46%, respectively (Table S1). Values for dDDH were <30% for all comparisons against strain ACI-7^T^ (Table S1). Both measures of OGRI fall well below the proposed species boundaries of 95–96% for ANI and 70% for dDDH [58,71, 72]. It is worth noting that genomic analyses of *M. arcticum* M2^T^ and *M. veterum* MK4^T^ revealed ANI and dDDH values (99.96% ANI, 99.9% dDDH) consistent with one single species. Since these are the type strains of these two species, this suggests that *M. arcticum* and *M. veterum* may be synonyms. However, a formal proposal would require confirmation of genome authenticity and validation of other data within their respective descriptions that is beyond the scope of this study.

### Taxonomic conclusions

The physiological characteristics differentiating strain ACI-7^T^ from the most phylogenetically related type species of *Methanobacterium* are summarized in Table 1. The isolate shared a similar temperature range and optimum with *M. oryzae* FPi^T^, *M. uliginosum* P2S^T^ and *M. arcticum* M2^T^ but differed considerably from *M. veterum* MK4^T^ and *M. movilense* MC-20^T^, which grow optimally at lower temperatures. All species including ACI-7,^T^ were able to use H_2_+CO_2_ as substrates, as is characteristic of the genus, but only ACI-7^T^, *M. oryzae* FPi^T^, *M. movilense* MC-20^T^ and *M. arcticum* M2^T^ are able to use formate. All species were neutrophilic, but strain ACI-7^T^ and *M. ivanovii* OCM 140^T^ are the most restricted in pH range for growth. Strain ACI-7^T^ tolerated similar NaCl concentrations as *M. oryzae* FPi^T^, both of which were more halotolerant than the other related species. Morphologically, cells of strain ACI-7^T^ were most similar to *M. uliginosum* P2S^T^ in size but formed filaments and aggregates as described for *M. oryzae* FPi^T^ and *M. bryantii* M.o.H.^T^. The formation of twisted chains by strain ACI-7^T^ (Fig. 1) is a distinct morphological trait not documented among its closest relatives and has only been described in one other *Methanobacterium* sp. (*M. aggregans* E09F.3^T^). Despite overall physiological similarity between strain ACI-7^T^ and its closest relative, *M. oryzae* FPi^T^, the two species are clearly differentiated by 16S rRNA gene similarity (97.09%), ANI (78.17%) and dDDH (22.0%). The most notable phenotypic differences between ACI-7^T^ and its other most highly related taxonomic relatives are its pH and temperature range as compared to *M. veterum* MK4^T^ (96.95% 16S rRNA, 75.33% ANI, 21.1% dDDH) and *M. arcticum* M2^T^ (96.47% 16S rRNA, 75.56% ANI, 21.1% dDDH) and the ability to use formate as a substrate compared to *M. bryantii* M.o.H.^T^ (96.68% 16S rRNA, 75.46% ANI, 21.1% dDDH) and *M. veterum* MK4^T^. Based on phenotypic differences, together with genomic and phylogenetic characteristics, strain ACI-7^T^ is considered to represent a novel species of the genus *Methanobacterium* for which the name *Methanobacterium nebraskense* sp. nov. is proposed.

**Table 1.** Differential characteristics of strain ACI-7^T^ and its closest phylogenetic relatives Strains: 1, ACI-7^T^; 2, *M. oryzae* FPi^T^ [25,26]; 3, *M. bryantii* M.o.H.^T^ [6,16, 73]; 4, *M. veterum* MK4^T^ [33]; 5, *M. ivanovii* OCM 140^T^ [22]; 6, *M. movilense* MC-20^T^ [25]; 7, *M. uliginosum* P2St^T^ [32,73]; 8, *M. arcticum* M2^T^ [13]; 9, *M. formicicum* MF^T^ [21,73]. Cells of all strains are non-motile rods. nd, not determined; +, positive; −, negative.

Characteristic	1	2	3	4	5	6	7	8	9
Source	Saline wetland soil	Rice field soil	Anaerobic digester	Permafrost sediment	Rock core	Subsurface lake	Marshy soil	Permafrost sediment	Anaerobic digester
Cell dimensions (width×length, µm)	0.2–0.4×0.9–4.2	0.3–0.4×3–10	0.5–1.0×10–15	0.4–0.45×2.0–8.0	0.5–0.8×1.2	0.6–0.7×3.5–4.0	0.2–0.6×1.9–3.9	0.45–0.5×3.0–6.0	0.4–0.8x2–15
Morphology									
Chains	+	+	+	+	+	+	+	+	+
Filaments	+	+	+	–	–	–	–	+	+
Aggregates	+	+	+	–	–	–	–	–	–
Temperature range (°C)	20–45	20–42	nd	10–46	nd	0–44	15–45	15–45	nd
Optimum temperature (°C)	40	40	37–39	28	45	33	40	37	30–45
pH range	6.5–8.5	6.0–8.5	nd	5.2–9.4	6.5–8.2	6.2–9.9	6.0–8.5	5.5–8.5	nd
Optimum pH	7.3	7.0	6.9–7.2	7.2–7.4	7.0–7.4	7.4	nd	6.8–7.2	7–7.5
NaCl range (g L^−1^)	0–25	0–25	nd	0–17.5	nd	0–17.5	nd	0–17.5	nd
Substrates									
H_2_+CO_2_	+	+	+	+	+	+	+	+	+
Formate	+	+	–	–	–	+	–	+	+
Alcohols	–	–	–	–	–	+	–	–	–
G+C content (mol%)^*^	31.8 (G)	31.4 (G)	33.2 (G)	33.2 (G)	36.6 (T_m_)	33.0 (LC)	29.4 (T_m_)	33.2 (G)	41–42 (BD)

*Determined by genome sequencing (G), HPLC (LC), buoyant density (BD), or thermal denaturation (T_m_).

## Description of *Methanobacterium nebraskense* sp. nov.

*Methanobacterium nebraskense* (ne.bras.ken′se. N.L. neut. adj. *nebraskense*, from Nebraska, USA, where the type strain was isolated).

Cells are non-motile rods, 0.9–4.2 µm in length and 0.2–0.4 µm in diameter, occurring singly, in chains, or as twisted chains and aggregates. Cells are strictly anaerobic, stain Gram-negative and resist lysis by 1% (w/v) SDS and hypotonic solution (distilled water). Colonies on solid medium are small (< 1 mm in diameter) and white with undefined margins, forming within 2 weeks of incubation. Utilizes H_2_+CO_2_ or formate for growth and methane formation, but not acetate, dimethylsulphide, 2-propanol+CO_2_, methanol, H_2_+methanol, methylamine, dimethylamine, trimethylamine or H_2_+methylamine. Growth factors present in yeast extract are required. Growth occurs from 20 to 45 °C (optimum 40 °C), at pH 6.5–8.5 (optimum 7.3) and 0–2.5% NaCl (optimum 0–1%).

The type strain, ACI-7^T^ (=DSM 118696^T^=ATCC TSD-487^T^), was isolated from a saline wetland located in eastern Nebraska, USA. The genomic G+C content of the type strain is 31.79 mol%, as determined by genome sequencing. The GenBank/ENA/DDBJ accession numbers for the 16S rRNA gene and complete genome are PQ144279 and CP166866, respectively.

## Supplementary material

10.1099/ijsem.0.007139 Uncited Supplementary Material 1.

## Abbreviations

ANI: average nucleotide identity
dDDH: digital DNA–DNA hybridization
DIC: differential interference contrast
GTDB: Genome Taxonomy Database
OGRI: overall genome relatedness index
