# Supplementary material for: Methanobacterium nebraskense sp. nov., a hydrogenotrophic methanogen isolated from saline wetland soil
Source: Int J Syst Evol Microbiol. 2026 Apr 21;76(4):007139. doi: 10.1099/ijsem.0.007139 (PMC13102254; doi:10.1099/ijsem.0.007139)
Supplement: Uncited Supplementary Material 1. [file ijsem-76-07139-s001.pdf]

Supplementary Information

Methanobacterium nebraskense sp. nov., a hydrogenotrophic methanogen isolated from saline wetland soil

Nicole A. Fiore[1,a], You Zhou[2], Karrie A. Weber[1,3,4*]

[1]School of Biological Sciences, University of Nebraska–Lincoln, Lincoln, NE, USA
[2]Center for Biotechnology, University of Nebraska–Lincoln, Lincoln, NE, USA
[3]Department of Earth and Atmospheric Sciences, University of Nebraska–Lincoln, Lincoln, NE, USA
[4]Daugherty Water for Food Institute, University of Nebraska, Lincoln, NE, USA

Current affiliations:
[a]Thayer School of Engineering, Dartmouth College, Hanover, NH, USA

[*]Address correspondence to Karrie A. Weber, kweber@unl.edu

Table S1. Strains and accession numbers for *Methanobacterium* species used in phylogenetic and genomic analyses and their respective values for 16S rRNA gene sequence similarity, average nucleotide identity (ANI), and digital DNA–DNA hybridization (dDDH) compared to strain ACI-7^{T}. NA, no available genome.

Species	16S rRNA			Genome			
	Strain	Accession no.	16S rRNA similarity (to ACI-7^{T})	Strain	Accession no.	ANI (vs. ACI-7^{T})	dDDH (vs. ACI-7^{T})
Methanobacterium aarhusense	H2-LR^{T}	NR_042895.1	95.27%	–	NA	–	–
Methanobacterium aggregans	E09F.3^{T}	NR_135896.1	95.66%	E09F.3^{T}	GCF_017874455.1	70.30%	20.5%
Methanobacterium alcaliphilum	WeN4^{T}	NR_028228.1	94.17%	WeN3	GCF_023227715.1	70.07%	19.1%
Methanobacterium alkalithermotolerans	CAN^{T}	KR349725.1	94.78%	CAN^{T}	GCF_018141185.1	69.22%	20.9%
Methanobacterium arcticum	M2^{T}	NR_115811.1	96.47%	M2^{T}	GCF_000746075.1	75.56%	21.1%
Methanobacterium aridiramus	CWC-01^{T}	MK979366.1	94.74%	CWC-01^{T}	GCF_030323845.1	68.45%	20.6%
Methanobacterium beijingense	8-2^{T}	NR_028202.1	95.05%	–	NA	–	–
Methanobacterium bryantii	M.o.H.^{T}	NR_042781.1	96.68%	M.o.H.^{T}	GCF_002287175.1	75.46%	21.1%
Methanobacterium congolense	C^{T}	NR_028175.1	95.78%	Buetzberg	GCF_900095295.1	70.61%	22.0%
Methanobacterium espanolae	GP9^{T}	NR_114483.1	96.57%	–	NA	–	–
Methanobacterium ferruginis	Mic6c05^{T}	NR_113045.1	94.60%	Mic6c05^{T}	GCF_030296715.1	69.66%	21.4%
Methanobacterium flexile	GH^{T}	NR_116276.1	93.91%	–	NA	–	–
Methanobacterium formicicum	MF^{T}	NR_115168.1	94.64%	Mb9	GCF_001458655.1	69.09%	20.7%
Methanobacterium ivanovii	OCM 140^{T}	NR_041716.1	96.86%	–	NA	–	–
Methanobacterium kanagiense	169^{T}	NR_112749.1	94.83%	–	NA	–	–
Methanobacterium lacus	17A1^{T}	NR_117917.1	95.25%	AL-21	GCF_000191585.1	70.06%	18.9%
Methanobacterium movens	TS-2^{T}	NR_116289.1	93.92%	TS-2^{T}	GCF_054656995.1	69.22%	20.9%
Methanobacterium movilense	MC-20^{T}	NR_133779.1	96.73%	–	NA	–	–
Methanobacterium oryzae	FPi^{T}	NR_028171.1	97.09%	FPi^{T}	GCF_050433505.1	78.17%	22.0%
Methanobacterium paludis	SWAN1^{T}	NR_133895.1	95.87%	SWAN1^{T}	GCF_000214725.1	70.92%	20.4%
Methanobacterium palustre	F^{T}	NR_114485.1	94.55%	–	NA	–	–
Methanobacterium petrolearium	Mic5c12^{T}	NR_113044.1	95.11%	Mic5c12^{T}	GCF_017873625.1	69.21%	25.7%
Methanobacterium spitsbergense	VT^{T}	OK037044.1	92.78%	VT^{T}	GCF_019931065.1	70.76%	19.6%
Methanobacterium subterraneum	A8p^{T}	NR_028247.1	95.18%	A8p^{T}	GCF_002813695.1	69.38%	21.5%
Methanobacterium thermaggregans	DSM 3266^{T}	NR_113572.1	92.29%	–	NA	–	–
Methanobacterium uliginosum	P2St^{T}	NR_104694.1	96.67%	–	NA	–	–
Methanobacterium veterum	MK4^{T}	NR_115935.1	96.95%	MK4^{T}	GCF_000745485.1	75.33%	21.1%

Table S2. Genes identified in the genome of strain ACI-7^T (accession no. CP166866) associated with the Wolfe cycle and archaeal-type Wood-Ljungdahl pathway and their chromosomal location (in bp, as denoted in the GenBank Record). EC, Enzyme Commission; KO, KEGG Orthology; H4MPT, tetrahydromethanopterin.

Enzyme	EC #	Gene	KO #	Copies	Location (GenBank Record)
formylmethanofurandehydrogenase	1.2.7.12	fwdA	K00200	1	382219..383979
		fwdB	K00201	1	384810..386108
		fwdC	K00202	1	383976..384788
		fwdD	K00203	1	381809..382201
		fwdE	K11261	1	complement(1547157..1547813)
		fwdF	K00205	2	380451..381563; complement(398349..399359)
		fwdG	K11260	1	381564..381809
		fwdH	K00204	1	379950..380432
formylmethanofuran/H4MPT formyltransferase	2.3.1.101	ftr	K00672	2	1603492..1604367; complement(885025..885918)
methenyl-H4MPT cyclohydrolase	3.5.4.27	mch	K01499	1	1348533..1349495
methylene-H4MPT dehydrogenase	1.5.98.1	mtd	K00319	1	639732..640562
methylene-H4MPT reductase	1.5.98.2	mer	K00320	1	complement(1146091..1147056)
methyl-H4MPT/coenzyme M methyltransferase	7.2.1.4	mtrA	K00577	1	946073..946789
		mtrB	K00578	1	945755..946060
		mtrC	K00579	1	944921..945742
		mtrD	K00580	1	944220..944921
		mtrE	K00581	1	943325..944206
		mtrF	K00582	1	946802..947008
		mtrG	K00583	1	947011..947253
		mtrH	K00584	1	947270..948223
methyl-coenzyme M reductase	2.8.4.1	mcrA	K00399	2	941622..943274; 1433188..1434840
		mcrB	K00401	2	938459..939787; 1430544..1431875
		mcrG	K00402	2	940855..941604; 1432392..1433186
electron-bifurcating hydrogenase-heterodisulfidereductase complex	1.8.98.5	mvhA	K14126	2	50284..51732; 959450..960868
		mvhD	K14127	2	810791..811219; 958071..958511
		mvhG	K14128	2	49364..50287; 958514..959449
		hdrA	K03388	1	518748..520730
		hdrB	K03389	1	96387..97322
		hdrC	K03390	1	95457..96374
F420-reducing hydrogenase	1.12.98.1	frhA	K00440	1	724520..725743
		frhB	K00441	2	137821..138870; 727068..727952
		frhG	K00443	1	726229..727056
energy-converting hydrogenase A		ehaA	K14092	1	1592657..1592965
		ehaB	K14093	1	1592962..1593465
		ehaC	K14094	1	1593531..1593779
		ehaD	K14095	1	1593798..1594085
		ehaE	K14096	1	1594078..1594338
		ehaF	K14097	1	1594335..1594856
		ehaG	K14098	1	1594811..1595539
		ehaH	K14099	1	1595578..1596255
		ehaI	K14100	1	1596277..1596492
		ehaJ	K14101	1	1596516..1597379
		ehaK	K14102	1	1597410..1597667
		ehaL	K14103	1	1597678..1598001
		ehaM	K14104	1	1597998..1598396
		ehaN	K14105	1	1598401..1598850
		ehaO	K14106	1	1598847..1599989
		ehaP	K14107	1	1600037..1601074
		ehaQ	K14108	1	1601071..1602432
		ehaR	K14109	1	1602462..1603487
carbon monoxide dehydrogenase/acetyl-CoA synthase	1.2.7.4; 2.3.1.169; 2.1.1.245	cdhA	K00192	1	1093656..1096013
		cdhB	K00195	1	1096024..1096533
		cdhC	K00193	1	1096579..1097964
		cdhD	K00194	1	1098771..1099934
		cdhE	K00197	1	1099947..1101320
